# Mammographic density changes during neoadjuvant breast cancer treatment: NeoDense, a prospective study in Sweden

**DOI:** 10.1016/j.breast.2020.05.013

**Published:** 2020-06-04

**Authors:** Ida Skarping, Daniel Förnvik, Uffe Heide-Jørgensen, Hanna Sartor, Per Hall, Sophia Zackrisson, Signe Borgquist

**Affiliations:** a Division of Oncology and Pathology, Department of Clinical Sciences, Lund University, Skåne University Hospital, Lund, Sweden; b Medical Radiation Physics, Department of Translational Medicine, Lund University, Skåne University Hospital, Malmö, Sweden; c Department of Clinical Epidemiology, Aarhus University Hospital, Aarhus, Denmark; d Diagnostic Radiology, Department of Translational Medicine, Lund University, Skåne University Hospital, Lund and Malmö, Sweden; e Department of Medical Epidemiology and Biostatistics, Karolinska Institute, Sweden; f Department of Oncology, Aarhus University Hospital, Aarhus, Denmark

**Keywords:** Breast cancer, Mammography, Breast density, Neoadjuvant therapy

## Abstract

**Objectives:**

To assess if mammographic density (MD) changes during neoadjuvant breast cancer treatment and is predictive of a pathological complete response (pCR).

**Methods:**

We prospectively included 200 breast cancer patients assigned to neoadjuvant chemotherapy (NACT) in the NeoDense study (2014–2019). Raw data mammograms were used to assess MD with a fully automated volumetric method and radiologists categorized MD using the Breast Imaging-Reporting and Data System (BI-RADS), 5th Edition. Logistic regression was used to calculate odds ratios (OR) for pCR comparing BI-RADS categories c vs. a, b, and d as well as with a 0.5% change in percent dense volume adjusting for baseline characteristics.

**Results:**

The overall median age was 53.1 years, and 48% of study participants were premenopausal pre-NACT. A total of 23% (N = 45) of the patients accomplished pCR following NACT. Patients with very dense breasts (BI-RADS d) were more likely to have a positive axillary lymph node status at diagnosis: 89% of the patients with very dense breasts compared to 72% in the entire cohort. A total of 74% of patients decreased their absolute dense volume during NACT. The likelihood of accomplishing pCR following NACT was independent of volumetric MD at diagnosis and change in volumetric MD during treatment. No trend was observed between decreasing density according to BI-RADS and the likelihood of accomplishing pCR following NACT.

**Conclusions:**

The majority of patients decreased their MD during NACT. We found no evidence of MD as a predictive marker of pCR in the neoadjuvant setting.

## Highlights

• A prospective study of breast cancer patients receiving neoadjuvant chemotherapy.
• Study patients with dense breasts often had positive axillary lymph nodes.
• Volumetric breast density decreased for most patients after neoadjuvant chemotherapy.
• We found no evidence of mammographic density as a predictive marker of pCR.

## Abbreviations

MD: mammographic density
BC: breast cancer
BI-RADS: Breast Imaging-Reporting and Data System
MRI: magnetic resonance imaging
NACT: neoadjuvant chemotherapy
pCR: pathological complete response
FEC: fluorouracil, epirubicin and cyclophosphamide
EC: epirubicin and cyclophosphamide
HER2: human epidermal growth factor receptor 2
ALN: axillary lymph node
ER: estrogen receptor
PR: progesterone receptor
VBD%: volumetric breast density percentage
FGV: fibroglandular volume
IQR: interquartile range
OR: odds ratio
BMI: body mass index
DCIS: ductal carcinoma in situ

## 1 Introduction

Mammographic density (MD) has gained significant interest and publicity in breast cancer (BC) screening. This is because women within the highest density categories have up to a 4- to 6-fold increased risk of primary BC in comparison to women with non-dense breasts [1]. The role of MD as a predictive marker in terms of response to diverse oncological treatments is less studied although it has been shown that a decrease in MD during tamoxifen treatment—both in the primary and secondary preventive setting—is associated with risk reduction for BC and recurrence hereof [2,3].

As a complement or alternative to the subjective Breast Imaging-Reporting and Data System (BI-RADS) categorization [4], assessment of MD can be estimated by one of many software products operating on both digital vendor-processed and unprocessed mammograms. Validated against BI-RADS and magnetic resonance imaging (MRI) [5,6], Volpara™ is robust and consistent across manufacturers [7,8] for measurement of volumetric MD.

On the tissue level, high MD represents a proliferative and pro-inflammatory environment [9,10]. It is plausible that the same biological mechanisms associated with tumor initiation and tumor growth in dense breasts may be responsible for a poorer treatment response. Previous studies including one from our group [11,12], have shown that patients with high MD are less responsive to neoadjuvant chemotherapy (NACT) in terms of pathological complete response (pCR)—a surrogate marker for long-term survival [13,14]. However, both previous studies were retrospective and used only a qualitative method for MD assessment (Wolfe categorization [15] and BI-RADS, respectively). Biomarkers, including imaging biomarkers, are needed for more personalized oncological treatment. This study aimed to investigate whether MD assessed with a volumetric quantitative method or a change in MD during NACT for BC is a predictive marker for pCR.

## 2 Material and methods

### 2.1 Cohort and clinical parameters

**Fig. 1:**
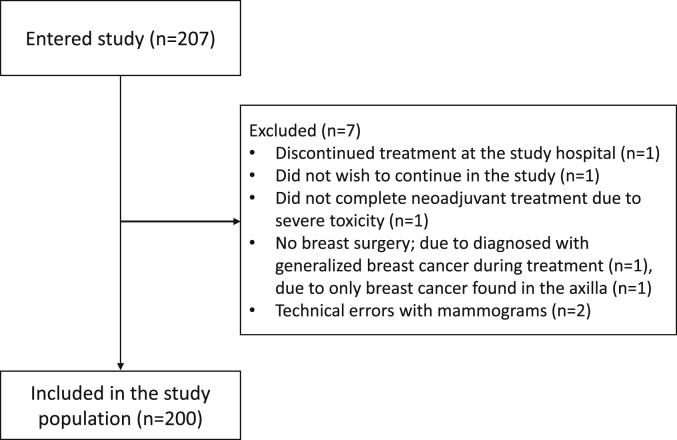
Patient flow chart.

**Fig. 2:**
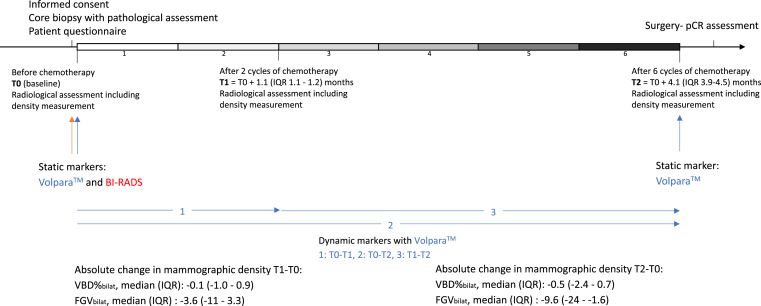
Study timeline.

From 2014 to 2019, we included 207 BC patients assigned to NACT within the ongoing SCAN-B trial (Clinical Trials ID NCT02306096) at Skåne University Hospital, Sweden [16,17]. Patients were enrolled at their first visit to the Department of Oncology following their BC diagnosis. The inclusion criteria were female, age ≥18 years, accepting NACT, and ability to give informed written consent. Reasons for exclusion (N = 7) are presented in Fig. 1. Bilateral mammograms and unilateral ultrasound of the cancerous breast and axilla were performed at baseline and after two and six cycles of chemotherapy, respectively (Fig. 2).

Patients received NACT according to the same guidelines and standard treatment included three series of fluorouracil, epirubicin, and cyclophosphamide (FEC) or epirubicin and cyclophosphamide (EC) followed by three series of docetaxel. HER2 double-blockade (trastuzumab and pertuzumab) was provided for human epidermal growth factor receptor 2 (HER2)-overexpression concomitantly with NACT. Ninety-seven percent of the patients received standard NACT, and 3% (N = 6) of the patients received a taxane-only NACT-regimen, and one patient received EC only. Among the patients with HER2-overexpressing tumors (N = 48), 94% received a double HER2-blockade whereas the remainder received only trastuzumab.

Clinical data and information on potential confounders were retrieved from patient questionnaires regarding anthropometrics, lifestyle factors, reproductive and hormonal history, previous breast disorders, and current and previous use of prespecified pharmaceuticals. Menopausal status at the time of diagnosis was defined according to self-reported menstrual history and patients with more than 1 year since the last period (secession of periods not caused by birth control, i.e., intrauterine hormonal contraceptive, or recent pregnancy/breastfeeding) were considered postmenopausal. Information on tumor characteristics was retrieved from clinical pathology reports. A pCR was defined as the absence of any residual invasive cancer in the resected breast after surgery as well as all sampled axillary lymph nodes (ALN) following completion of NACT [18]. For the four patients with bilateral BC, the breast with the largest tumor/tumors was followed and evaluated. The Research Electronic Data Capture application was used for secure data entry [19]. The study was approved by the Regional Ethics Committee in Lund, Sweden (committee’s reference numbers: 2014/13, 2014/521, and 2016/521).

### 2.2 Digital mammography

Through prospectively collected radiological study forms (Supplementary Material 1), detailed radiological tumor characteristics were retrieved and noted in real-time at the examination. Clinical bilateral digital mammograms in three views were acquired on different machines: GE Senographe Pristina (3%), Philips MammoDiagnost DR (17%), Philips MicroDose (2%), and Siemens Mammomat Inspiration (77%). All images were saved in their raw, unprocessed format, and MD was estimated with the computerized fully-automatic software Volpara™ (version 1.5.4.0, Volpara Solutions Limited, Wellington, New Zealand) for which technical details are described elsewhere [20]. Briefly, the volumetric estimate is derived from a 2-dimensional digital mammogram that creates an artificial volume based on assumptions of the anatomy of the breast, knowledge of the breast thickness, and image processing [20]. Volumetric breast density percentage (VBD%) is a continuous variable calculated as the ratio of absolute dense tissue volume [fibroglandular volume (FGV)] to total breast volume. At each time point, the craniocaudal view and the mediolateral oblique view in both breasts and the contralateral healthy breast only, respectively, were used to calculate MD (VBD% and FGV). In line with a previous study showing good concordance in MD between the ipsilateral tumorous breast and the contralateral healthy breast [21], a simplified validation was performed showing no large difference in volumetric MD in cancer affected and non-affected breast supporting the use of the average VBD%_bilat_ in the descriptive statistics. Experienced breast radiologists, in direct connection to the examination, assessed the MD of the contralateral breast according to BI-RADS 5th edition [4].

### 2.3 Statistical analysis

We first plotted the cumulative distribution of the mean of the VBD% in both breasts (VBD%_bilat_) within each BI-RADS level. We also plotted the change in VBD%_bilat_ from baseline to T1 (after 2nd chemotherapy cycle) and from baseline to T2 (after 6th chemotherapy cycle) versus baseline VBD%_bilat_; equivalent plots were made with the mean of FGV in both breasts (FGV_bilat_) instead of VBD%_bilat_.

Next, patient characteristics were summarized by the BI-RADS level at baseline. Categorical variables were described by counts and percentages whereas continuous variables were described by their median and interquartile range (IQR). For categorical variables, we furthermore assessed the median and IQR of baseline VBD%_bilat_ within each level of the variable. Finally, we described the baseline characteristics and VBD%_bilat_ at T1 and T2 of the patients by pCR status at the end of the follow-up.

We then set up logistic regression models including either VBD%_bilat_, the VBD% of the contralateral non-cancer affected breast only (VBD%_contra_), FGV_bilat_, or BI-RADS as the independent variable. pCR was the dependent (outcome) variable. We also considered dynamic models, i.e., models in which absolute change in MD from T0 to T1 [i.e., VBD% (at T1) minus VBD% (at T0)], T0 to T2, and T1 to T2, respectively, served as independent variables. For both VBD%_bilat_ and VBD%_contra_, we established models with an odds ratio (OR) corresponding to a 0.3, 0.5, and 2.0 percentage point change in VBD%, respectively. In addition, models based on relative change (OR corresponding to 5% change) in VBD%_bilat_ as the independent variable were established. For FGV_bilat_, we built the models with an OR corresponding to a 1- and 3-unit change, respectively. In the logistic regression models, we used generalized estimating equations to consider within-hospital site correlations. We set up both crude models and partially- and fully adjusted models. In the partially adjusted models, we included age, body mass index (BMI), menopausal status, parity and hormone replacement therapy; in the fully adjusted models, we also included ER, Ki67, HER2, ALN status, and tumor size at diagnosis. In the dynamic models, we also adjusted for MD at baseline and T1 because a decrease in MD was mostly seen in patients with high MD at baseline. Finally, similar logistic regression models were used to analyze the cohorts within subgroups defined by ALN, ER, and menopausal status. All analyses were carried out in SAS (SAS Institute Inc., Version 9.4, Cary, NC, USA).

## 3 Results

The distribution of baseline characteristics according to BI-RADS and VBD%_bilat_ is presented in Table 1 for the 200 BC patients receiving NACT (Fig. 1). For the whole cohort, the median age was 53.1 years (IQR 45.9 to 62.5), the median BMI was 25.6 (IQR 22.4 to 28.7), median VBD%_bilat_ at diagnosis was 11.0 (IQR 7.5 to 17.1), and median FGV_bilat_ was 73.5 cm^3^ (IQR 52.4 to 100).

**Table 1:** Patient and tumor characteristics according to mammographic density at diagnosis.

			BI-RADS a (N = 9)	BI-RADS b (N = 74)	BI-RADS c (N = 90)	BI-RADS d (N = 27)	VBD%_bilat_ median (IQR)
Age at diagnosis	Median (IQR)		62 (58–67)	56 (46–65)	51 (44–62)	47 (43–60)	
BMI	Median (IQR)		34.0 (28.7–36.8)	26.5 (22.4–28.7)	24.8 (22.3–28.7)	23.9 (22.1–25.6)	
Age at menarche	Median (IQR)		13 (11–14)	13 (12–14)	13 (12–14)	13 (12–13)	
	Missing	N = 5	0 (0.0)	1 (1.4)	4 (4.4)	0 (0.0)	
Menopausal status	Premenopausal	N = 95	0 (0)	28 (37.8)	51 (56.7)	16 (59.3)	14.0 (10.0–19.6)
	Postmenopausal	N = 105	9 (100)	46 (62.2)	39 (43.3)	11 (40.7)	8.3 (5.7–12.6)
Number of pregnancies	None	N = 19	0 (0)	6 (8.1)	10 (11.1)	3 (11.1)	12.0 (6.0–20.1)
	1	N = 28	1 (11.1)	11 (14.9)	10 (11.1)	6 (22.2)	10.7 (8.5–17.8)
	2	N = 76	0 (0)	26 (35.1)	40 (44.4)	10 (37.0)	12.4 (7.7–17.8)
	3+	N = 77	8 (88.9)	31 (41.9)	30 (33.3)	8 (29.6)	9.8 (6.9–15.0)
Any live birth	No	N = 31	1 (11.1)	9 (12.2)	15 (16.7)	6 (22.2)	12.8 (7.1–18.5)
	Yes	N = 169	8 (88.9)	65 (87.8)	75 (83.3)	21 (77.8)	11.0 (7.6–16.7)
Age first birth (years)	No children	N = 31	1 (11.1)	9 (12.2)	15 (16.7)	6 (22.2)	12.8 (7.1–18.5)
	<20	N = 10	2 (22.2)	4 (5.4)	4 (4.4)	0 (0)	8.6 (5.8–10.0)
	20–29	N = 90	6 (66.7)	33 (44.6)	40 (44.4)	11 (40.7)	10.1 (6.6–16.9)
	30–34	N = 44	0 (0)	15 (20.3)	22 (24.4)	7 (25.9)	13.3 (9.6–19.0)
	35+	N = 21	0 (0)	11 (14.9)	7 (7.8)	3 (11.1)	10.0 (8.7–15.6)
	Missing	N = 4	0 (0)	2 (2.7)	2 (2.2)	0 (0)	8.5 (6.9–10.4)
Number of biological children	None	N = 31	1 (11.1)	9 (12.2)	15 (16.7)	6 (22.2)	12.8 (7.1–18.5)
	1	N = 34	0 (0)	16 (21.6)	14 (15.6)	4 (14.8)	9.5 (7.9–12.7)
	2	N = 96	3 (33.3)	33 (44.6)	47 (52.2)	13 (48.1)	11.7 (7.7–17.4)
	3+	N = 39	5 (55.6)	16 (21.6)	14 (15.6)	4 (14.8)	9.6 (6.4–16.7)
Alcohol use once a week or more often	Yes	N = 92	3 (33.3)	34 (45.9)	42 (46.7)	13 (48.1)	11.2 (7.7–18.0)
	No	N = 107	6 (66.7)	40 (54.1)	47 (52.2)	14 (51.9)	10.4 (7.2–16.5)
	Missing	N = 1	0 (0)	0 (0)	1 (1.1)	0 (0)	17.1 (17.1–17.1)
Exercise	More than 4 h/week	N = 64	2 (22.2)	23 (31.1)	31 (34.4)	8 (29.6)	12.0 (7.9–15.7)
	Less than 4 h/week	N = 100	5 (55.6)	34 (45.9)	44 (48.9)	17 (63.0)	11.8 (7.6–18.9)
	Nothing	N = 34	2 (22.2)	16 (21.6)	14 (15.6)	2 (7.4)	8.9 (6.1–11.8)
	Missing	N = 2	0 (0)	1 (1.4)	1 (1.1)	0 (0)	10.0 (5.7–14.3)
Smoking	Current	N = 19	2 (22.2)	8 (10.8)	8 (8.9)	1 (3.7)	8.7 (5.8–10.8)
	Former	N = 67	3 (33.3)	24 (32.4)	30 (33.3)	10 (37.0)	10.1 (6.4–16.6)
	Never	N = 114	4 (44.4)	42 (56.8)	52 (57.8)	16 (59.3)	12.3 (8.3–17.5)
Ever hormone replacement therapy	Yes	N = 18	0 (0)	7 (9.5)	8 (8.9)	3 (11.1)	11.0 (8.6–18.4)
	No	N = 182	9 (100)	67 (90.5)	82 (91.1)	24 (88.9)	11.1 (7.4–16.9)
Oral contraceptives	Current	N = 5	0 (0)	1 (1.4)	2 (2.2)	2 (7.4)	14.1 (13.1–14.3)
	Former	N = 146	5 (55.6)	50 (67.6)	72 (80.0)	19 (70.4)	12.3 (7.9–18.0)
	Never	N = 48	4 (44.4)	22 (29.7)	16 (17.8)	6 (22.2)	8.7 (5.9–12.0)
	Missing	N = 1	0 (0)	1 (1.4)	0 (0)	0 (0)	5.7 (5.7–5.7)
Tumor size at diagnosis (mm)^a^	Median (IQR)		34 (26–40)	27 (21–38)	30 (21–40)	36 (23–42)	
	Missing	N = 3	0 (0.0)	1 (1.4)	2 (2.2)	0 (0.0)	
Estrogen receptor status	Positive (≥10%)	N = 121	5 (55.6)	45 (60.8)	52 (57.8)	19 (70.4)	11.2 (7.6–16.7)
	Negative (<10%)	N = 79	4 (44.4)	29 (39.2)	38 (42.2)	8 (29.6)	10.9 (7.3–18.3)
Progesterone receptor status	Positive (≥10%)	N = 103	6 (66.7)	38 (51.4)	43 (47.8)	16 (59.3)	10.8 (7.5–16.6)
	Negative (<10%)	N = 96	3 (33.3)	36 (48.6)	46 (51.1)	11 (40.7)	12.0 (7.3–18.3)
	Missing	N = 1	0 (0)	0 (0)	1 (1.1)	0 (0)	10.1 (10.1–10.1)
HER2 receptor status^b^	Positive	N = 48	4 (44.4)	19 (25.7)	19 (21.1)	6 (22.2)	10.0 (6.8–17.4)
	Negative	N = 152	5 (55.6)	55 (74.3)	71 (78.9)	21 (77.8)	11.3 (7.6–16.7)
Ki67^c^	High	N = 157	8 (88.9)	60 (81.1)	69 (76.7)	20 (74.1)	10.6 (7.3–16.9)
	Intermediate	N = 30	1 (11.1)	7 (9.5)	15 (16.7)	7 (25.9)	14.7 (9.2–19.6)
	Low	N = 11	0 (0)	6 (8.1)	5 (5.6)	0 (0)	10.1 (9.0–14.6)
	Missing	N = 2	0 (0)	1 (1.4)	1 (1.1)	0 (0)	8.8 (7.5–10.1)
Axillary lymph node status	Positive	N = 143	6 (66.7)	52 (70.3)	61 (67.8)	24 (88.9)	10.8 (7.3–17.1)
	Negative	N = 57	3 (33.3)	22 (29.7)	29 (32.2)	3 (11.1)	11.7 (7.6–17.1)

a Tumor size (largest diameter) was retrieved from study specific radiological protocols and when the size assessments varied between the modalities, the largest measurement was used.

b If the tumor was assessed as 3+ with immunohistochemistry and/or amplified with *in situ* hybridization.

c Tumors were considered as low, intermediate or highly proliferative according to laboratory specific cutoffs (site 1: low 0–20%; intermediate 21–30%; high 31–100%, site 2: low 0–14%; intermediate 15–24%; high 25–100%) for proportion of cells staining positive for Ki67.

Patients being younger, premenopausal, leaner (a lower BMI), nulliparous and/or having a history of oral contraceptive use had higher median VBD%_bilat_ at baseline in comparison to their opposites (for age and BMI, respectively, visual assessment was done of boxplot for two groups divided by the median).

In comparison to patients with less dense breasts, patients with very dense breast (BI-RADS d, N = 27) were more likely to have ER-positive tumors and to have a positive ALN status at diagnosis (89%), but VBD%_bilat_ was similar regardless of ER expression and ALN status. In total, only a few tumors had low proliferation [Ki67, (N = 11)]. None of the patients categorized as BI-RADS d (N = 27) had low proliferative tumors. Except for BI-RADS a, there was a trend in that denser breasts implied larger tumors.

Patients with pCR following NACT (N = 45) compared to patients without pCR (N = 155) had similar VBD%_bilat_ at all three time points (Table 2). Patients with ER-negative, PR-negative, and/or HER2-overexpressing tumors, negative ALN status, or high proliferation (Ki67) were more likely to obtain pCR irrespective of MD.

**Table 2:** Patient and tumor characteristics at diagnosis according to pathological complete response (pCR).

		pCR (N = 45)	Non-pCR (N = 155)
VBD%_bilat_ diagnosis	Median (IQR)	12.4 (7.1–17.1)	11.0 (7.7–16.9)
	Missing	2 (4.4)	5 (3.2)
VBD%_bilat_ at T1	Median (IQR)	10.9 (7.1–17.0)	10.7 (7.8–15.9)
	Missing	1 (2.2)	7 (4.5)
VBD%_bilat_ at T2	Median (IQR)	11.2 (7.3–15.3)	9.7 (7.7–14.7)
	Missing	1 (2.2)	6 (3.9)
BI-RADS at baseline	a	3 (6.7)	6 (3.9)
	b	19 (42.2)	55 (35.5)
	c	17 (37.8)	73 (47.1)
	d	6 (13.3)	21 (13.5)
Age at diagnosis	Median (IQR)	53 (46–62)	53 (46–63)
BMI	Median (IQR)	25.5 (22.9–28.7)	25.6 (22.4–28.7)
Age at menarche	Median (IQR)	13 (12–14)	13 (12–14)
	Missing	1 (2.2)	4 (2.6)
Menopausal status	Premenopausal	20 (44.4)	75 (48.4)
	Postmenopausal	25 (55.6)	80 (51.6)
Number of pregnancies	None	3 (6.7)	16 (10.3)
	1	9 (20.0)	19 (12.3)
	2	13 (28.9)	63 (40.6)
	3+	20 (44.4)	57 (36.8)
Any live birth	No	6 (13.3)	25 (16.1)
	Yes	39 (86.7)	130 (83.9)
Age first birth (years)	No children	6 (13.3)	25 (16.1)
	<20	4 (8.9)	6 (3.9)
	20–29	18 (40.0)	72 (46.5)
	30–34	11 (24.4)	33 (21.3)
	35+	6 (13.3)	15 (9.7)
	Missing	0 (0)	4 (2.6)
Number of biological children	None	6 (13.3)	25 (16.1)
	1	9 (20.0)	25 (16.1)
	2	19 (42.2)	77 (49.7)
	3+	11 (24.4)	28 (18.1)
Alcohol use once a week or more often	Yes	18 (40.0)	74 (47.7)
	No	26 (57.8)	81 (52.3)
	Missing	1 (2.2)	0 (0)
Exercise	More than 4 h/week	11 (24.4)	53 (34.2)
	Less than 4 h/week	26 (57.8)	74 (47.7)
	Nothing	8 (17.8)	26 (16.8)
	Missing	0 (0)	2 (1.3)
Smoking	Current	4 (8.9)	15 (9.7)
	Former	16 (35.6)	51 (32.9)
	Never	25 (55.6)	89 (57.4)
Ever hormone replacement therapy	Yes	2 (4.4)	16 (10.3)
	No	43 (95.6)	139 (89.7)
Oral contraceptives	Current	0 (0)	5 (3.2)
	Former	35 (77.8)	111 (71.6)
	Never	10 (22.2)	38 (24.5)
	Missing	0 (0)	1 (0.6)
Tumor size at diagnosis (mm)^a^	Median (IQR)	29 (22–38)	30 (21–40)
	Missing	1 (2.2)	2 (1.3)
Estrogen receptor status	Positive (≥10%)	10 (22.2)	111 (71.6)
	Negative (<10%)	35 (77.8)	44 (28.4)
Progesterone receptor status	Positive (≥10%)	5 (11.1)	98 (63.2)
	Negative (<10%)	40 (88.9)	56 (36.1)
	Missing	0 (0)	1 (0.6)
HER2 receptor status^b^	Positive	20 (44.4)	28 (18.1)
	Negative	25 (55.6)	127 (81.9)
Ki67^c^	High	40 (88.9)	117 (75.5)
	Intermediate	5 (11.1)	25 (16.1)
	Low	0 (0)	11 (7.1)
	Missing	0 (0)	2 (1.3)
Axillary node status	Positive	25 (55.6)	118 (76.1)
	Negative	20 (44.4)	37 (23.9)

a Tumor size (largest diameter) was retrieved from study specific radiological protocols and when the size assessments varied between the modalities, the largest measurement was used.

b If the tumor was assessed as 3+ with immunohistochemistry and/or amplified with *in situ* hybridization.

c Tumors were considered as low, intermediate or highly proliferative according to laboratory specific cutoffs (site 1: low 0–20%; intermediate 21–30%; high 31–100%, site 2: low 0–14%; intermediate 15–24%; high 25–100%) for proportion of cells staining positive for Ki67.

**Fig. 3:**
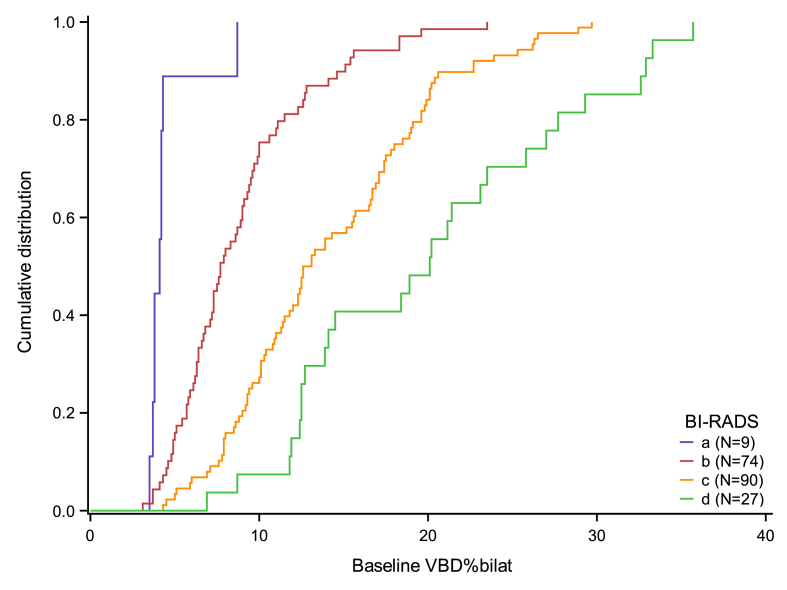
Agreement between BI-RADS and volumetric breast density percentage (VBD%_bilat_).

The distribution of BI-RADS categories in relation to VBD%_bilat_ measured with Volpara™ at baseline is visualized in Fig. 3.

About half of the patients (47%) decreased their VBD%_bilat_ between baseline and T1 and the corresponding percentage between baseline and T2 was 56%. Only a small temporal change in VBD%_bilat_ was seen between baseline and T1 [median absolute decrease −0.1 (IQR -1.0 to 0.9)] whereas a slightly more pronounced change in VBD%_bilat_ was seen between baseline and T2 [median absolute decrease −0.5 (IQR -2.4 to 0.7)]. A larger proportion of patients decreased their FGV_bilat_ during NACT; a total of 61% of the patients decreased their FGV_bilat_ between baseline and T1 [median absolute decrease −3.6 (IQR -11 to 3.3)] and 74% of the patients decreased their FGV_bilat_ between baseline and T2 [median absolute decrease −9.6 (IQR -24 to −1.6)] (Supplementary Material 2).

No association was seen between MD measured with Volpara™ as a static marker at T0 and T2 (VBD%_bilat_, VBD%_contra_, and FGV_bilat_) or as a dynamic marker (ΔVBD%_bilat_, ΔVBD%_contra_, and ΔFGV_bilat_) and pCR using different logistic regression models, iteratively adjusted for increasing numbers of variables (Table 3, Supplementary Material 3, and Supplementary Material 4). Furthermore, no association was found between volumetric MD and pCR for OR corresponding to 0.3 and 2.0 percentage point change in VBD%, respectively, 5% change in VBD%_bilat_, and a 1-unit change in FGV. We did not find any association between ΔVBD%_bilat_, ΔVBD%_contra_, or ΔFGV_bilat_ in the subgroup analyses based on menopausal status, ER expression, and ALN status. No trend was observed between decreasing BI-RADS categories and the likelihood of accomplishing pCR (Table 4). When using BI-RADS c as a reference, patients with both lower and higher BI-RADS categories had a higher likelihood of achieving pCR.

**Table 3:** Associations between VBD%_bilat_ and pathological complete response following neoadjuvant chemotherapy.

VBD%_bilat_ exposure type, OR correspond to a 0.5 unit change in VBD%_bilat_	N	Cases	Model 1 OR (95% CI)	Model 2 OR (95% CI)	Model 3 OR (95% CI)	Model 3 adjusted for VBD%_bilat_ at T0 OR (95% CI)
Static T0	188	42	1.00 (0.98–1.03)	1.00 (0.97–1.03)	1.01 (0.97–1.06)	
Static T2	187	43	1.00 (0.98–1.03)	1.00 (0.97–1.04)	1.01 (0.97–1.06)	
Dynamic T0-T1	180	41	1.00 (0.91–1.09)	1.00 (0.92–1.08)	0.96 (0.87–1.06)	0.97 (0.89–1.07)
Dynamic T0-T2	181	41	1.02 (0.94–1.10)	1.02 (0.94–1.10)	0.99 (0.91–1.08)	1.00 (0.92–1.09)
Dynamic T1-T2	181	42	1.02 (0.94–1.11)	1.02 (0.94–1.11)	1.02 (0.93–1.12)	1.05 (0.95–1.16)^a^

Model 1: crude analysis.

Model 2: minimally adjusted (age, BMI, menopause, parity, HRT) analysis.

Model 3: fully adjusted (model 2 + ER, Ki67, HER2, axillary node status and tumor size at diagnosis) analysis.

a Adjusted for VBD%_bilat_ at T1.

**Table 4:** Associations between BI-RADS at diagnosis and pathological complete response following neoadjuvant chemotherapy.

BI-RADS	N	Cases	Model 1 OR (95% CI)	Model 2 OR (95% CI)	Model 3 OR (95% CI)
a	9	3	2.22 (1.49–3.30)	2.32 (1.09–4.94)	1.56 (0.43–5.70)
b	72	19	1.59 (1.46–1.73)	1.57 (1.37–1.80)	1.49 (1.45–1.52)
c	87	16			
d	27	6	1.27 (0.34–4.75)	1.23 (0.37–4.11)	2.37 (1.15–4.88)

Model 1: crude analysis.

Model 2: minimally adjusted (age, BMI, menopause, parity, HRT) analysis.

Model 3: fully adjusted (model 2 + ER, Ki67, HER2, axillary node status and tumor size at diagnosis) analysis.

## 4 Discussion

In this study of 200 prospectively included BC patients, approximately three-quarters of the patients decreased their FGV_bilat_ during NACT. We found no evidence of MD as a predictive marker in the neoadjuvant setting (neither with Volpara™ nor with BI-RADS). Two previous studies [11,12] found low MD at diagnosis associated with improved rates of pCR, however, both were retrospective and used a qualitative density method. Patient, tumor, and treatment characteristics were comparable across the previous two studies as well as this work (besides the single HER2-blockade in contrast to the double HER2-blockade in the current study). Another retrospective study using BI-RADS for MD assessment did not find such an association [22]; however, it was based on a cohort that was different from many others—a low pCR rate (15%), suboptimal NACT (i.e., no anti-HER2 treatment to patients with HER2-overexpressing tumors), and a pCR definition that included patients with residual invasive tumor cells making comparison with other studies difficult. To the best of our knowledge, this is the first study to investigate the association between MD measured with a volumetric quantitative method and response to NACT, and investigate the rate and quantification of MD change during NACT.

It is of interest to look at the temporal association between MD and a certain intervention since changes in MD can modulate the risk, and recurrence, of BC [2,23]. While a larger group of studies [2,3,[24], [25], [26], [27], [28]] have explored the effect of endocrine treatment on MD, less is known about the association between treatment response to chemotherapy (with or without anti-HER2 therapy) and MD. A longitudinal study investigating the effect of antiestrogen treatment in the adjuvant BC setting on volumetric MD changes in a relatively large study cohort showed an annual decrease in VBD% of 0–2% [24]. The corresponding number for a small study using MRI was almost 4% [28].

Chen et al. further investigated the change in breast density measured with MRI during NACT in a small number of patients (N < 45 in both studies) and showed an 11–13% reduction in percent breast density measured with MRI [29,30]. Previous studies demonstrate a reduction in MD during adjuvant chemotherapy [[31], [32], [33]], however only one of them provided a quantitative measure of the change in MD (−2.9 percentage points %MD). In two studies, women, predominantly younger women, with ≥10% MD reduction had a reduced risk of contralateral BC compared to women with less reduced MD [31,33]. In our study, the median decline in MD during NACT was −0.5 percentage points (IQR -2.4 to 0.7) correlating to a mean decline of 4.5%. In this context, despite our relatively short period of time between first and last measurement (4.1 months, IQR 3.9–4.5 months), we should have been able to detect and quantify a potential association between density and outcome measure (pCR).

MD changes throughout a woman’s life along with age and hormonal events [34] with a steep decline occurring around menopausal change [35]. In the NSABP B-30 trial [36], the vast majority of premenopausal patients receiving adjuvant chemotherapy for BC had at least a 6-month long period of amenorrhea, and it is reasonable to expect similar proportions in our neoadjuvant-treated cohort since the patients were treated with the same combination of chemotherapy agents [36]. There was a more pronounced association between MD reductions and chemotherapy in premenopausal patients in comparison to postmenopausal patients [29,32]: This is likely related to a change in the hormonal milieu. Also, lobular atrophies may contribute to a MD reduction during chemotherapy [37]. Thus, it is difficult to identify the underlying biological explanation for the small decline in MD seen in our study.

Bilateral and contralateral mammograms, respectively, were used for Volpara™-assessment in this study. Each Volpara™-output includes VBD%, FGV, and the absolute non-dense volume in the breast/breasts. Previous studies have shown a positive association between both FGV and VBD% and BC risk with a more pronounced association seen with VBD% [[38], [39], [40]]; these data indicate the importance of the microenvironment of the non-dense breast tissue in the BC etiology. Tumor characteristics as well as host factors influence the tumor response to treatment, e.g. triple negative subtypes are known to be highly responsive to NACT [41]. This motivates the adjustments in our logistic regression models. Representing the microenvironment of the surrounding breast tissue [22], MD is a host factor that influences the tumor response to treatment. In terms of MD and tumor characteristics, previous studies have shown associations between higher MD and positive ALN and larger tumor size [[42], [43], [44]]. In our study, approximately 70% of the patients had a positive ALN—the corresponding number for patients with very dense breasts (BI-RADS d, N = 27) was 89%. In our cohort, the median tumor size was 30.0 mm (IQR 22.0–40.0 mm) with a tendency for a larger tumor, the denser the breast. One plausible explanation contributing to the inconsistent results regarding MD as a predictive marker for pCR during NACT seen in our studies is that, in the current study, a high MD is seemingly associated with high proliferation (Ki67), which is in turn associated with a better response to NACT [[45], [46], [47]]. This dilutes the previously suggested association between MD and pCR.

Several systems for pathological evaluation of the complex post-NACT response exist, and the clinical importance of residual ductal carcinoma *in situ* (DCIS) only is not yet fully understood [48]. Regardless of whether residual DCIS only is considered as pCR or not, both definitions are associated with similar improved prognosis [14], but the pCR rates are lower in studies using the most conservative definition. In order to include all patients with favorable prognosis, in this study, patients with only residual DCIS were categorized as having accomplished pCR.

Our study has several strengths including the prospective cohort with detailed information on patient and tumor characteristics. We used both a fully-automated volumetric density method on raw digital mammograms as well as BI-RADS categorization of processed images. Previous studies have shown different degrees of agreement and correlation [49] between VBD% and BI-RADS ranging from poor to good [[50], [51], [52]]. Given the proportions of the displayed patient and tumor characteristics and the ratio of pCR, we suggest that our cohort is a good reflection of the general patient group as a whole and offers external validity.

The issue of lacking consistency regarding the vendor and model of the machines must be addressed. We made no adjustment for this variable because Volpara™ has been shown to offer a consistent measurement of volumetric MD across vendors [7,8]. The matter of alignment [53] of mammograms that makes the amount of breast tissue similar in each image must be brought to attention when dealing with a change in MD over time. To minimize error due to alignment, each technician was repeatedly instructed to similarly position the breast each time and to capture the entire breast and not just the tumor. Thus, we believe that the principally important concept of alignment will not affect our results on a group level. No subgroup analyses based on the St. Gallen BC subtype [54] were performed due to our limited number of patients. However, when stratifying on ER expression, no association was seen between volumetric MD and pCR. A larger dataset is needed to better understand the role of MD as a predictive marker during NACT in different subtypes of BC. This enables clinical applicability. Longer follow-up might be needed to demonstrate a consistent decline in MD.

## 5 Conclusion

In summary, a large proportion of the patients decreased their mammographic density during neoadjuvant chemotherapy for breast cancer. We found no evidence of mammographic density, assessed with both quantitative and qualitative methods, as a predictive marker for complete pathological response in the neoadjuvant setting. Future larger studies should examine whether mammographic density holds predictive value regarding treatment with chemotherapy.

## Data availability

The datasets used and/or analyzed during the current study are available from the corresponding author on reasonable request.

## Authors’ contributions

IS participated in designing the study, made the study protocols, coordinated the enrollment of patients, collected the data, wrote the statistical plan, interpreted the data, solely drafted the manuscript (except for “Statistical analyses”), and coordinated the revision of the manuscript. DF provided technical support during image collection, aided in density assessments, interpreted the data, and revised the manuscript. UH assisted in making the statistical plan, performed the statistical analysis, interpreted the data, co-wrote the “Statistical analyses” part of the manuscript, and substantially revised the manuscript. HS intellectually contributed to decisions regarding density assessment and interpretation and revised the manuscript. PH participated in the design of the study, provided technical support during image collection, interpreted the data, and revised the manuscript. SZ participated in the general design of the study, interpreted the data, and contributed to the adjustment of the manuscript. SB was the main contributor to the initial design of the study, interpreted the data, and revised the manuscript. All authors have read and approved the final manuscript.

## Authors’ information

IS: MD, Physician at Skåne University Hospital, Lund, PhD-student Oncology, Lund University, Skåne University Hospital Lund, Sweden.

DF: Medical Physicist, PhD, Medical Radiation Physics, Department of Translational Medicine, Lund University, Skåne University Hospital, Malmö, Sweden.

UH: MSc, PhD, Statistician in the Department of Clinical Epidemiology, Aarhus University Hospital, Aarhus, Denmark.

HS: MD, Specialist in Radiology, PhD, Associate Professor, Diagnostic Radiology, Department of Translational Medicine, Lund University, Skåne University Hospital Lund and Malmö, Sweden.

PH: MD, PhD, full professor at the Department of Medical Epidemiology and Biostatistics, Karolinska Institutet, Sweden.

SZ: MD, PhD, Professor, Senior Consultant, Department of Imaging and Functional Medicine, Skåne University Hospital Malmö, and Diagnostic Radiology, Department of Translational Medicine, Lund University, Sweden.

SB: MD, PhD, Professor, Consultant, Department of Oncology, Aarhus University Hospital, Aarhus, Denmark. Visiting Professor, Division of Oncology and Pathology, Lund University, Sweden.

## Funding

This work was supported by grants from the Swedish Breast Cancer Group (BRO) and the Governmental Funding of Clinical Research within National Health Services, Sweden (ALF-medel). The Volpara™ software was provided by the Volpara™ company. The funding resources had no role in the study design, data collection, analyses, data interpretation, writing of the manuscript, or the decision to submit the manuscript for publication.

## Ethical approval

All procedures performed in studies involving human participants were in accordance with the ethical standards of the institutional and/or national research committee and with the 1964 Helsinki Declaration and its later amendments or comparable ethical standards. The study was approved by the Regional Ethics Committee in Lund, Sweden (committee’s reference number: 2014/13, 2014/521 and 2016/521).

## Informed consent

Oral and written information was provided to the patients. Informed written consent was obtained from all individual participants included in the study.

## Declaration of competing interest

SZ and HS have received speakers’ fees and travel support from Siemens Healthcare AG. SZ has received consultancy fees from Collective Minds Radiology AB. PH is a member of a scientific advisory board for: Cancer Research UK, iCAD and Atossa Genetics. SB has received speakers’ fees from Pfizer, is a member of a Pfizer advisory board, and has received travel support from Roche. The other authors declare that they have no competing interests.
