# Supplementary material for: Mammographic density changes during neoadjuvant breast cancer treatment: NeoDense, a prospective study in Sweden
Source: Breast. 2020 Jun 4;53:33–41. doi: 10.1016/j.breast.2020.05.013 (PMC7375568; doi:10.1016/j.breast.2020.05.013)
Supplement: Multimedia component 1 [file mmc1.pdf]

NeoDense

Skånes Onkologiska Klinik

”Före kemo”, ”Efter cykel 2”, ”Efter kemo”

**Födelsedatum: 19_ _ _ _ _ _**	**Studie nr:**		**Patientinitialer:**
Sjukhus	☐ Lund	☐Malmö	☐Helsingborg

Instruktioner till röntgenläkare: Vänligen svara på samtliga frågor för varje undersökning vid varje undersökningstillfälle. Vid oklarheter kontakta studieansvarig sjuksköterska och skriv i kommentarrutor.

OBS! För 1 års uppföljning vänligen använd annat formulär.

Tidpunkt för undersökning	☐ 1: Före kemo ☐ 2: Efter cykel 2<br>☐ 3: Efter kemo
Undersökningsdatum	20_ _ _ _ _ _
Namn på rtg-läkare (den som fyller i protokollet)	
Bilateral cancer	☐ Nej ☐ Ja (om ja: använd 2 separata formulär)
Bröstcancer sida	☐ Höger ☐ Vänster
Undersökning<br>(alla fyra skall vara genomförda vid varje undersökningstillfälle)	☐ Mammografi cancerbröst<br>☐ Mammografi kontralateralt bröst<br>☐ Ultraljud cancerbröst<br>☐ Ultraljud ipsilateral axill
Tilläggsundersökningar MALMÖ	☐ Tomosyntes cancerbröst<br>☐ Tomosyntes kontralateralt bröst

Studieansvarig ssk: Lina Zander (040-33 74 61) **Studieansvariga läkare**:Ida Skarping (ida.skarping@med.lu.se), Hanna Sartor (040-338984), Sophia Zackrisson (040-338797), Signe Borgquist (signe.borgquist@med.lu.se)
Studieansvarig sjukhusfysiker: Daniel Förnvik (0706 08 40 40)

NeoDense

Skånes Onkologiska Klinik

”Före kemo”, ”Efter cykel 2”, ”Efter kemo”

**Födelsedatum: 19_ _ _ _ _ _ _**	**Studie nr:**	**Patientinitialer:**	
Sjukhus	□Lund	□Malmö	□Helsingborg
Tidpunkt för undersökning	□1: Före kemo<br>□3: Efter kemo	□2: Efter cykel 2	

MAMMOGRAFI

Cancerbröst

TUMÖR

Antal tumörer	nr:_____ □ Ej mätbart
Vid multifokalitet ange resp. diameter utifrån tumörernas storleksordning vid diagnostillfället.	
1:a tumörens största diameter (individuella tumören)	____mm Vy: □ MLO □ CC □ ML
2:a tumörens största diameter	____mm
3:e tumörens största diameter	____mm
Kommentar	
Tumörens växtsätt	□ Välavgränsad 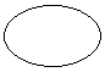<br>□ Strålig 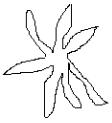<br>□ Diffus 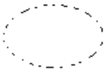<br>□ Maligna förkalkningar
MG kod	□ 1 □ 2 □ 3 □ 4 □ 5

HELA BRÖSTVÄVNADEN- Kontralateralt bröst

Bröstdensitet (enligt BI-RADS 5th Edition)	□ a) Brösten innehåller nästan enbart fettvävnad<br>□ b) Det finns utspridda områden med tät körtelvävnad<br>□ c) Brösten är oregelbundet täta, vilket kan skymma små förändringar<br>□ d) Brösten är väldigt täta, vilket minskar mammografins sensitivitet

Studieansvarig ssk: Lina Zander (040-33 74 61) **Studieansvariga läkare**:Ida Skarping (ida.skarping@med.lu.se), Hanna Sartor (040-338984), Sophia Zackrisson (040-338797), Signe Borgquist (signe.borgquist@med.lu.se)
Studieansvarig sjukhusfysiker: Daniel Förnvik (0706 08 40 40)

NeoDense

Skånes Onkologiska Klinik
”Före kemo”, ”Efter cykel 2”, ”Efter kemo”

**Födelsedatum: 19_ _ _ _ _ _**	**Studie nr:**	**Patientinitialer:**
Sjukhus	□Lund	□Malmö	□Helsingborg
Tidpunkt för undersökning	□1: Före kemo □2: Efter cykel 2<br>□3: Efter kemo	

ULTRALJUD

Cancerbröst

TUMÖR

Antal tumörer	nr:_____ □ Ej mätbart
Vid multifokalitet ange resp. diameter utifrån tumörernas storleksordning vid diagnostillfället.	
1:a tumörens största diameter (individuella tumören)	____mm
2:a tumörens största diameter	____mm
3:e tumörens största diameter	____mm
Kommentar	
Ekogenicitet största tumör	□ Låg □ Varierande □ Hög
U kod	□ 1 □2 □3 □4 □ 5

AXILLÄRA LYMFKÖRTLAR

Antal patologiska lymfkörtlar	□ 0 □ 1 □2 □≥3
Största patologiska lymfkörtellängd	Kortaxel: _____mm<br>Långaxel: _____mm □ Ej mätbart
Ekogenicitet lymfkörtel	□ Låg □ Varierande □ Hög
Kommentar	

Studieansvarig ssk: Lina Zander (040-33 74 61) **Studieansvariga läkare**:Ida Skarping (ida.skarping@med.lu.se), Hanna Sartor (040-338984), Sophia Zackrisson (040-338797), Signe Borgquist (signe.borgquist@med.lu.se)
Studieansvarig sjukhusfysiker: Daniel Förnvik (0706 08 40 40)

NeoDense

Skånes Onkologiska Klinik

”Före kemo”, ”Efter cykel 2”, ”Efter kemo”

**Födelsedatum: 19_ _ _ _ _ _ _**	**Studie nr:**	**Patientinitialer:**	
Sjukhus	□Lund	□Malmö	□ Helsingborg
Tidpunkt för undersökning	□1: Före kemo<br>□3: Efter kemo	□2: Efter cykel 2	

Endast MALMÖ
TOMOSYNTES

Cancerbröst

TUMÖR

Antal tumörer	nr:_____ □ Ej mätbart
Vid multifokalitet ange resp. diameter utifrån tumörernas storleksordning vid diagnostillfället.	
1:a tumörens största diameter (individuella tumören)	____mm
2:a tumörens största diameter	____mm
3:e tumörens största diameter	____mm
Kommentar	
Tumörens växtsätt	□ Välavgränsad 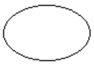<br>□ Strålig 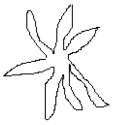<br>□ Diffus 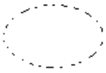<br>□ Maligna förkalkningar
MG kod	□ 1 □ 2 □ 3 □ 4 □ 5

Övriga kommentarer

Tack för din medverkan!
