# Supplementary figures and images for: Mammographic density changes during neoadjuvant breast cancer treatment: NeoDense, a prospective study in Sweden
Source: Breast. 2020 Jun 4;53:33–41. doi: 10.1016/j.breast.2020.05.013 (PMC7375568; doi:10.1016/j.breast.2020.05.013)

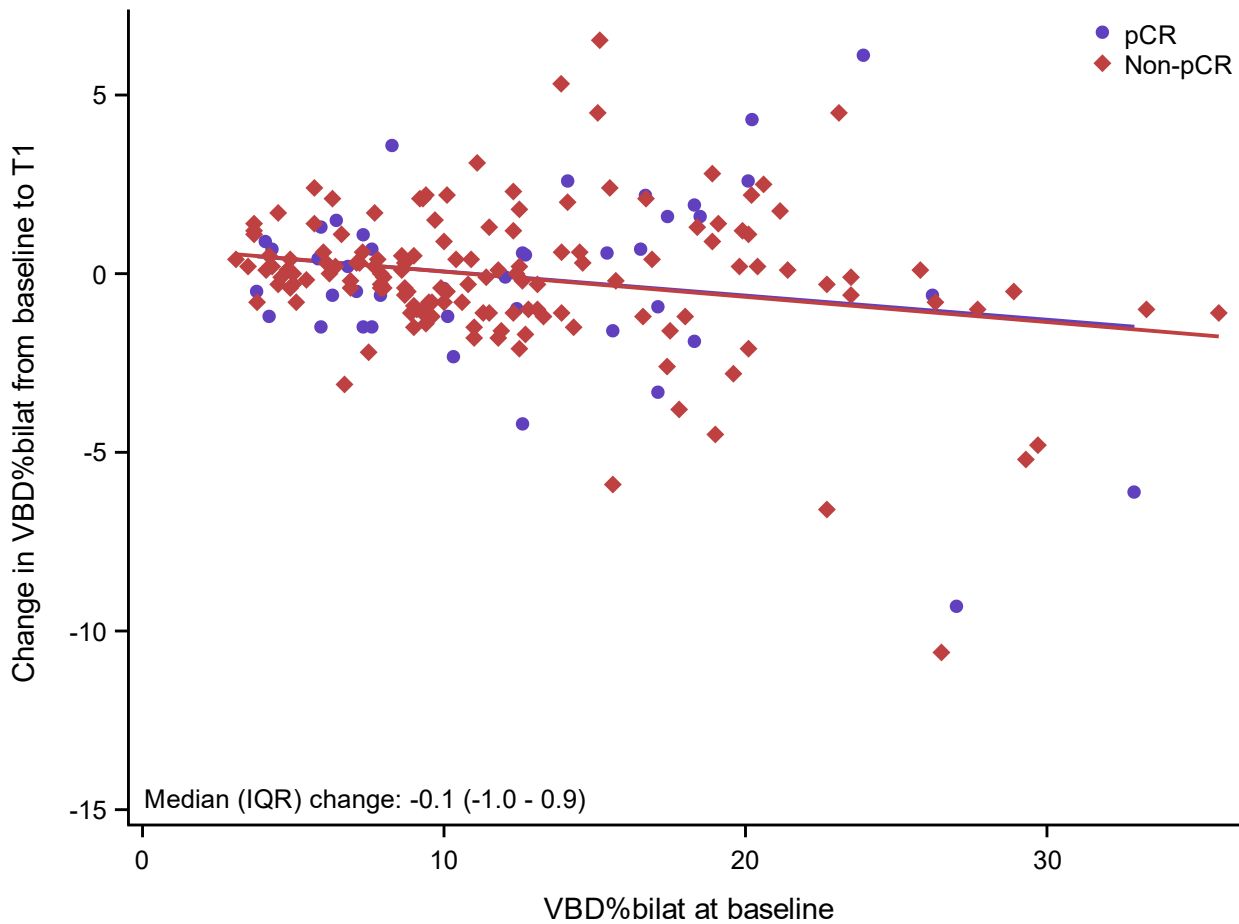

Supplement: Multimedia component 2 [file mmc2.zip › ybrst_3052_Supplementary Material 2 a_spl_.pdf]

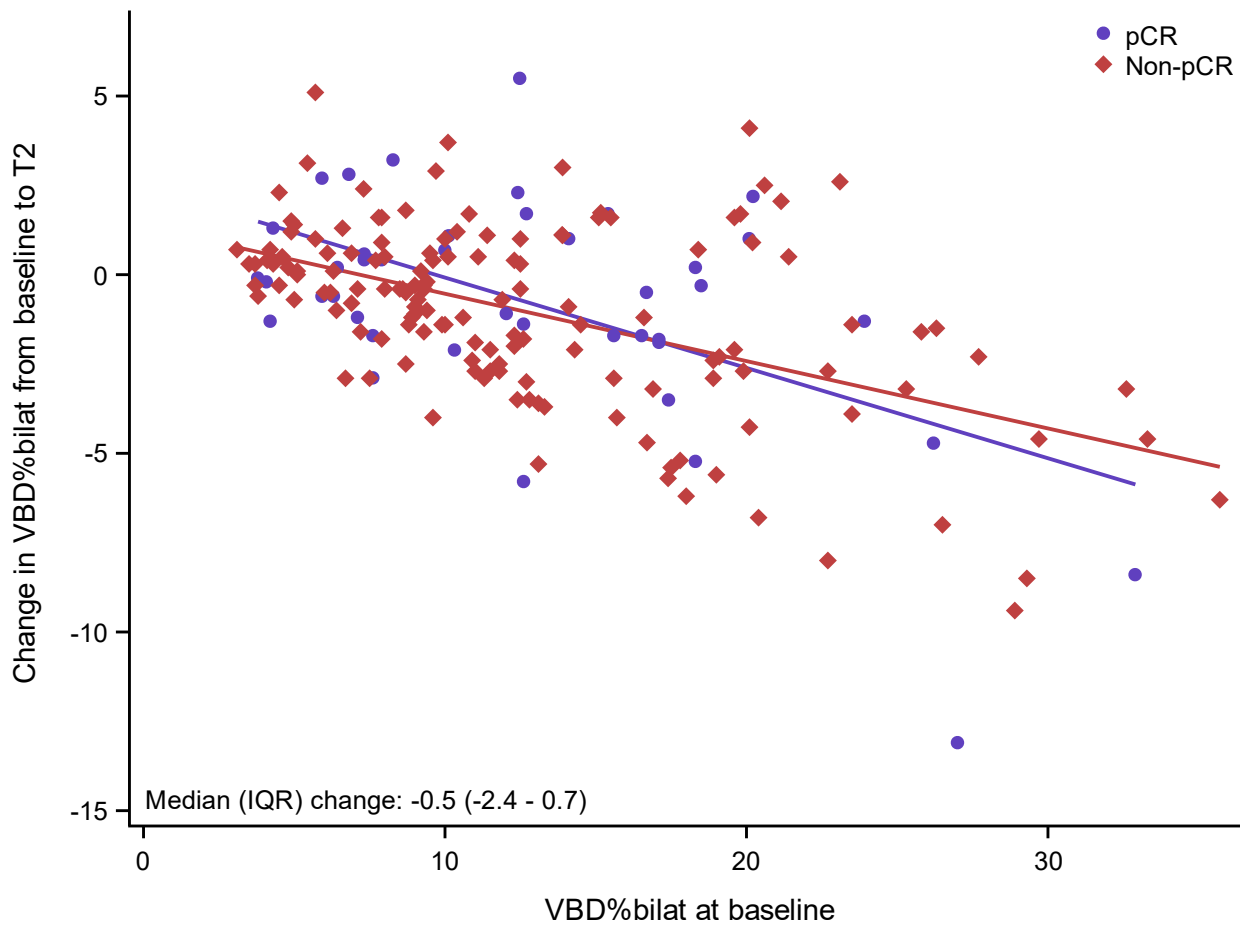

Supplement: Multimedia component 2 [file mmc2.zip › ybrst_3052_Supplementary Material 2 b_spl_.pdf]

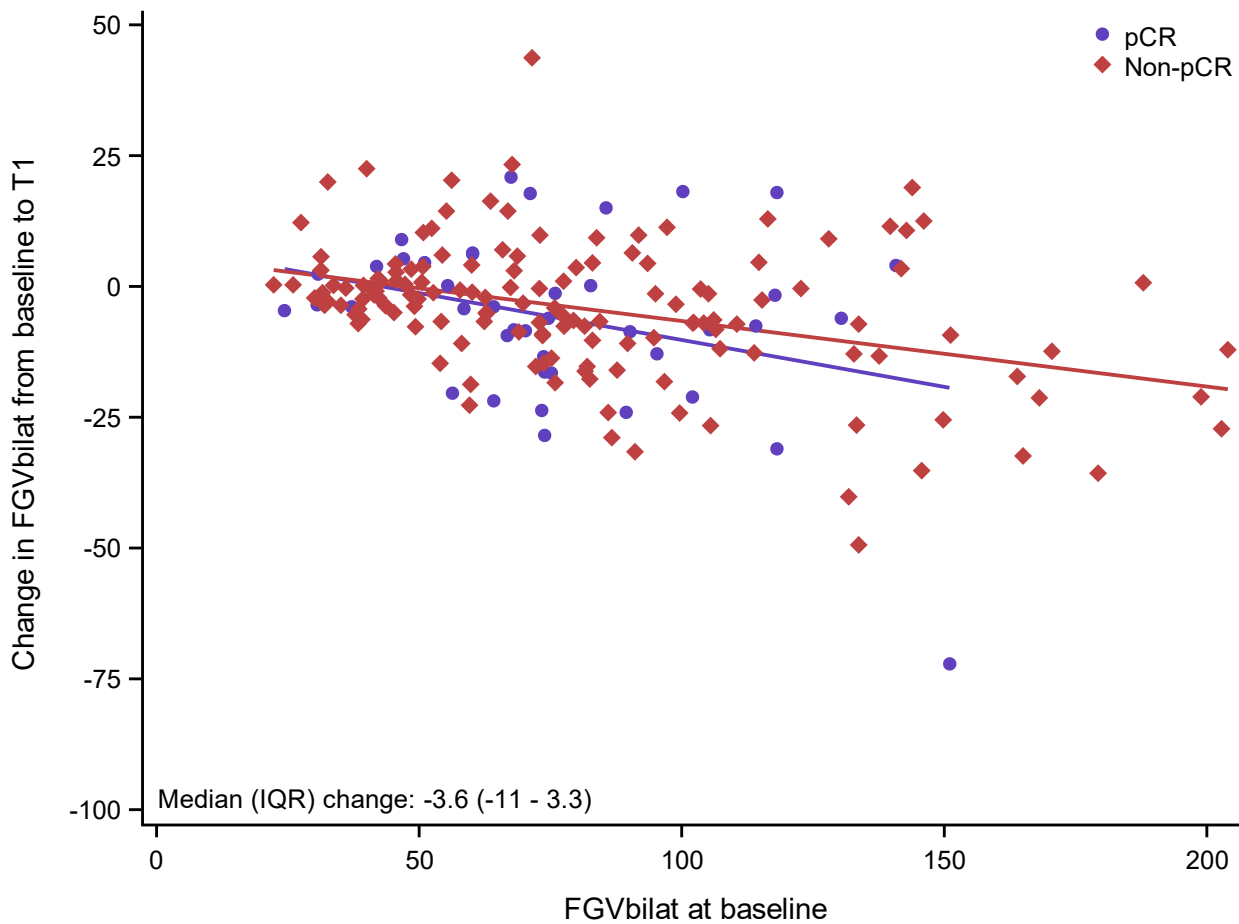

Supplement: Multimedia component 2 [file mmc2.zip › ybrst_3052_Supplementary Material 2 c_spl_.pdf]

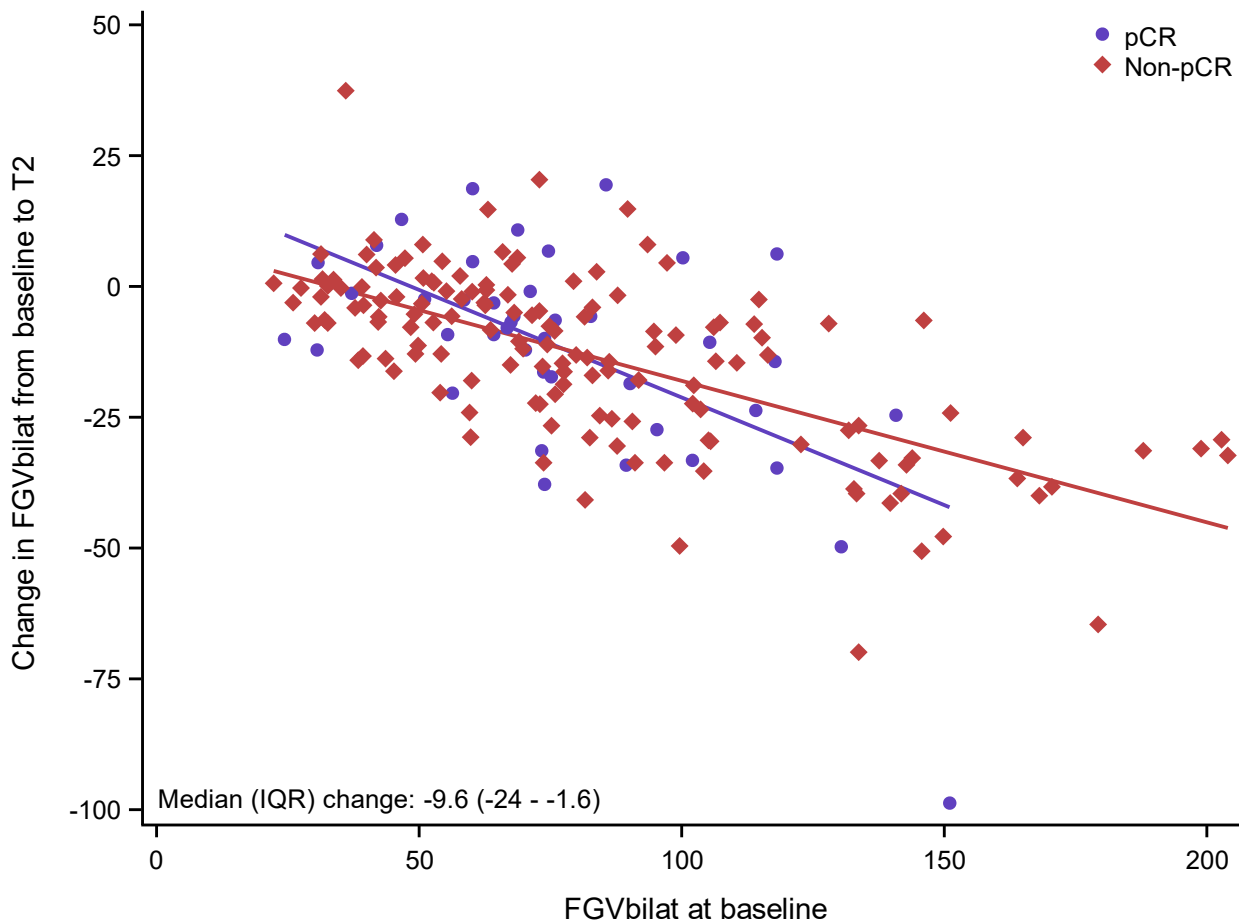

Supplement: Multimedia component 2 [file mmc2.zip › ybrst_3052_Supplementary Material 2 d_spl_.pdf]
