# Supplementary material for: Mammographic density changes during neoadjuvant breast cancer treatment: NeoDense, a prospective study in Sweden
Source: Breast. 2020 Jun 4;53:33–41. doi: 10.1016/j.breast.2020.05.013 (PMC7375568; doi:10.1016/j.breast.2020.05.013)
Supplement: Multimedia component 3 [file mmc3.rtf]

Supplementary material 3. Associations between VBD%contra and pathological complete response following neoadjuvant chemotherapy
VBD%contra exposure type, OR correspond to a 0.5 unit change in VBD%contra	N	Cases	Model 1 OR (95% CI)	Model 2 OR (95% CI) 	Model 3 OR (95% CI) 	Model 3 adjusted for VBD%contra at T0 OR (95% CI)	
Static T0	184	42	1.00 (0.98 - 1.03)	1.00 (0.97 - 1.03)	1.01 (0.97 - 1.04)		
Static T2	186	43	1.00 (0.98 - 1.02)	1.00 (0.98 - 1.03)	1.01 (0.97 - 1.05)		
Dynamic T0-T1	176	41	1.02 (0.93 - 1.12)	1.02 (0.93 - 1.11)	0.99 (0.87 - 1.13)	0.99 (0.88 - 1.12)	
Dynamic T0-T2	175	41	1.05 (0.96 - 1.14)	1.05 (0.96 - 1.15)	1.03 (0.93 - 1.14)	1.04 (0.93 - 1.17)	
Dynamic T1-T2	177	42	1.03 (0.96 - 1.11)	1.03 (0.96 - 1.12)	1.03 (0.95 - 1.13)	*1.07 (0.97 - 1.19)	

	Model 1:  crude analysis 
	Model 2:  minimally adjusted (age, BMI, menopause, parity, HRT) analysis 
	Model 3:  fully adjusted (model 2 + ER, Ki67, HER2, axillary node status and tumor size at diagnosis) analysis
	*adjusted for VBD%contra at T1
