# Supplementary material for: Mammographic density changes during neoadjuvant breast cancer treatment: NeoDense, a prospective study in Sweden
Source: Breast. 2020 Jun 4;53:33–41. doi: 10.1016/j.breast.2020.05.013 (PMC7375568; doi:10.1016/j.breast.2020.05.013)
Supplement: Multimedia component 4 [file mmc4.rtf]

Supplementary material 4. Associations between FGVbilat and pathological complete response following neoadjuvant chemotherapy
FGVbilat exposure type, OR correspond to a 3 unit change in FGVbilat	N	Cases	Model 1 OR (95% CI)	Model 1 OR (95% CI) 	Model 3 OR (95% CI) 	Model 3 adjusted for FGVbilat at T0 OR (95% CI)	
Static T0	188	42	0.99 (0.96 - 1.01)	0.99 (0.96 - 1.01)	1.00 (0.96 - 1.04)		
Static T2	187	43	0.99 (0.96 - 1.01)	0.99 (0.96 - 1.02)	1.00 (0.96 - 1.04)		
Dynamic T0-T1	180	41	0.98 (0.90 - 1.05)	0.97 (0.89 - 1.04)	0.93 (0.83 - 1.05)	0.93 (0.82 - 1.05)	
Dynamic T0-T2	181	41	1.02 (0.95 - 1.10)	1.02 (0.94 - 1.10)	0.95 (0.83 - 1.08)	0.94 (0.81 - 1.08)	
Dynamic T1-T2	181	42	1.05 (0.97 - 1.13)	1.04 (0.96 - 1.14)	0.98 (0.89 - 1.09)	*0.98 (0.87 - 1.09)	

	Model 1:  crude analysis 
	Model 2:  minimally adjusted (age, BMI, menopause, parity, HRT) analysis 
	Model 3:  fully adjusted (model 2 + ER, Ki67, HER2, axillary node status and tumor size at diagnosis) analysis
	*adjusted for FGVbilat at T1
